# Global Sexual Fertility in the Opportunistic Pathogen *Aspergillus fumigatus* and Identification of New Supermater Strains

**DOI:** 10.3390/jof6040258

**Published:** 2020-10-30

**Authors:** Sameira S. Swilaiman, Céline M. O’Gorman, Wenyue Du, Janyce A. Sugui, Joanne Del Buono, Matthias Brock, Kyung J. Kwon-Chung, George Szakacs, Paul S. Dyer

**Affiliations:** 1 School of Life Sciences, University of Nottingham, University Park, Nottingham NG7 2RD, UK; samiraswar@yahoo.com (S.S.S.); celine.tevlin@gmail.com (C.M.O.); mbxwd@exmail.nottingham.ac.uk (W.D.); Joanne.Del_Buono@nottingham.ac.uk (J.D.B.); mbzmmb@exmail.nottingham.ac.uk (M.B.); 2 Molecular Microbiology Section, Laboratory of Clinical Immunology and Microbiology, National Institute of Allergy and Infectious Diseases, National Institutes of Health, Bethesda, MD 20825, USA; janyce.janyce.janyce@gmail.com (J.A.S.); jkchung@niaid.nih.gov (K.J.K.-C.); 3 Department of Applied Biotechnology and Food Science, Budapest University of Technology and Economics, Szent Gellert ter 4, 1111 Budapest, Hungary; szakacs_g@hotmail.com

**Keywords:** *Aspergillus fumigatus*, sexual fertility, cleistothecia, supermater

## Abstract

A sexual cycle in *Aspergillus fumigatus* was first described in 2009 with isolates from Dublin, Ireland. However, the extent to which worldwide isolates can undergo sexual reproduction has remained unclear. In this study a global collection of 131 isolates was established with a near 1:1 ratio of mating types. All isolates were crossed to *MAT1-1* or *MAT1-2* Irish strains, and a subset of isolates from different continents were crossed together. Ninety seven percent of isolates were found to produce cleistothecia with at least one mating partner, showing that sexual fertility is not limited to the Irish population but is a characteristic of global *A. fumigatus*. However, large variation was seen in numbers of cleistothecia produced per cross, suggesting differences in the possibility for genetic exchange between strains in nature. The majority of crosses produced ascospores with >50% germination rates, but with wide variation evident. A high temperature heat shock was required to induce ascospore germination. Finally, a new set of highly fertile *MAT1-1* and *MAT1-2* supermater strains were identified and pyrimidine auxotrophs generated for community use. Results provide insights into the potential for the *A. fumigatus* sexual cycle to generate genetic variation and allow gene flow of medically important traits.

## 1. Introduction

*Aspergillus fumigatus* is a cosmopolitan saprotrophic fungus which plays an important role in the decomposition of organic matter and recycling of carbon and nitrogen, with soil and rotting vegetation being natural ecological niches [1,2]. It is one of the most common microorganisms found in compost and is also commonly found in human habitats, e.g., pillows [3,4]. The species is also one of the most important opportunistic fungal pathogens of humans. Inhalation of asexual conidia rarely shows adverse effects in immune-competent hosts [2,5]. However, when the host immune response is either too strong or too weak a spectrum of diseases collectively termed aspergillosis can occur [6,7,8]. These can be divided into major forms according to the site of infection and degree of colonisation, and include severe asthma with fungal sensitization (SAFS), allergic bronchial pulmonary aspergillosis (ABPA), chronic pulmonary aspergillosis (CPA), invasive pulmonary aspergillosis (IPA), and invasive bronchial aspergillosis (IBA) [7,9,10]. Infections by *A. fumigatus* in immune-compromised hosts can result in mortality rates reaching 95% in certain situations [11,12]. The high mortality rate of *A. fumigatus* appears to be due to a combination of a weakened immune response by the host, the virulence of the microorganism itself and probably delays in establishing a diagnosis, which can decrease the success of treatments [13]. This situation has been made worse by the clinical and environmental evolution of resistance to triazole antifungals, which are used to treat the disease [14,15]. Most recently *A. fumigatus* infections have been reported as a complication of COVID19 cases [16,17,18].

As with many other clinically important fungal species, *A. fumigatus* has traditionally been considered as an asexual organism. However, a major breakthrough was reported in 2009 with discovery of a sexual reproductive cycle in *A. fumigatus,* which resulted in the production of cleistothecia and ascospores [19]. The sexual cycle was induced by pairing strains of opposite mating type on oatmeal agar at 30 °C and leaving them to incubate for six to twelve months. This discovery followed on from previous work showing evidence for sexuality in *A. fumigatus*. In particular, earlier genome analysis had revealed that the species contained an apparently functional complement of sex-related genes, and associated experimental work showed expression of several of these genes [20,21,22]. This included, most notably, the expression of mating-type (*MAT*) genes, which are key regulators of sexual reproduction [23]. Isolates of *A. fumigatus* present in nature were shown to contain either *MAT1-1* or *MAT1-2* idiomorphs, consistent with a heterothallic (obligate outbreeding) sexual breeding system. Furthermore, strains of opposite *MAT1-1* or *MAT1-2* mating types were found to be present at a near 1:1 ratio in a global survey of isolates, and could be found in close proximity to each other where local sites were sampled [19,22].

The discovery of sexual reproduction in *A. fumigatus* is of great potential medical significance for various reasons. Subsequent work confirmed that the fungus exhibits Mendelian inheritance of genetic traits, with recombination and cross over demonstrated during the sexual cycle [24]. Furthermore, classical crossing experiments revealed monogenic inheritance for TR_34_/L98H azole drug resistance, demonstrating exchange of genetic material as a result of sexual reproduction [25]. Therefore, the presence of a sexual cycle can allow gene flow in populations and result in progeny with increased virulence and/or increased resistance to antifungal treatments [26]. Indeed, there is accumulating evidence that the sexual cycle is linked with the evolution and spread of resistance to azole antifungals [15,25,27]. Sexual spores are also often better at surviving harsh environmental conditions [28,29]. In addition, the sexual cycle can confound diagnostic tests based on the hypothesis of clonality [26].

The breakthrough work of O’Gorman et al. [19] reported an investigation of sexuality and successful crossings in only a small sample of environmental *A. fumigatus* isolates from a population around Dublin, Ireland. There have since been reports of sexual crossing in a limited number of isolates from other worldwide locations, including the identification of supermater strains, which were able to complete the sexual cycle in only four weeks [24,25,30]. However, the extent to which global isolates of *A. fumigatus* are in general able to reproduce sexually has remained unknown. This is an important question, as this will provide an indication of the risk of spread of resistance to antifungal drugs via sexual recombination given evidence elsewhere of a very limited capacity for sexual reproduction in other fungal pathogens, which appear to be evolving towards asexuality [31].

The present study, therefore, reports the establishment of a worldwide collection of isolates of *A. fumigatus* and its screening for mating type, and then determination of the sexual fertility of these isolates as judged by the ability to form cleistothecia. Furthermore, the percentage of viable ascospores formed in crosses was assessed together with an investigation of factors influencing germination of sexual ascospores. Finally, the detection of additional supermater strains of *A. fumigatus* with high sexual fertility, combined with the production of pyrimidine auxotrophic strains for community use, is described.

## 2. Materials and Methods

### 2.1. Strains and Growth Conditions

A number of *Aspergillus fumigatus* isolates were obtained from existing stocks in the University of Nottingham Botany Department (BDUN) collection [22]. In addition, further new isolates were obtained from worldwide environmental locations by the use of soil dilution plate methods using incubation at 37 ℃ or 45 ℃ on either 2% malt extract agar (MEA; Oxoid, Basingstoke, UK) or potato dextrose agar (PDA; Oxoid, Basingstoke, UK) plates supplemented either with 100 mg/L oxytetracycline dihydrate or 100 mg/L doxycycline hyclate [32,33]. *A. fumigatus*-like colonies were further purified by streaking on MEA or PDA plates supplemented with 0.05–0.1% Triton X100 (a colony-size-reducing agent) at 37 °C. Additional clinical isolates were obtained as previously described [34] or via procedures recommended by UK Standards for Microbiology Investigations (https://www.gov.uk/government/collections/standards-for-microbiology-investigations-smi).

Isolates were provisionally identified as *A. fumigatus* on the basis of morphological characteristics, and identity was confirmed, if necessary, by sequencing of a portion of the beta-tubulin gene [35,36]. Long-term stocks were then established by either lyophilisation or storage in 10% glycerol under liquid nitrogen to prevent any loss of sexual fertility due to repeated vegetative subculture [26]. Strains were thereafter routinely maintained on an *Aspergillus* complete medium (ACM [21]) or on MEA at 28 °C–30 °C.

### 2.2. DNA Extraction, PCR Mating-Type Diagnostic and RAPD-PCR Analysis

Cultures were grown and genomic DNA extracted as described by Swilaiman et al. [37] using either a phenol/chloroform protocol or DNeasy Plant Mini kit (Qiagen, Manchester, UK). A multiplex polymerase chain reaction (PCR) mating-type assay was then used to determine the mating-type identity (*MAT1-1* or *MAT1-2*) of each isolate using the diagnostic primer set AFM1 (CCTTGACGCGATGGGGTGG), AFM2 (CGCTCCTCATCAGAACAACTCG) and AFM3 (CGGAAATCTGATGTCGCCACG) according to Paoletti et al. [22]. RAPD-PCR was used to assess clonality of isolates, using six primers (R151, OPAX16, UBC90, OPW08, OPAJ05, RC08 [38]) according to O’Gorman et al. [19].

### 2.3. Crossing of A. fumigatus Isolates and Sexual Fertility

Conidia were collected from two-month old cultures grown at room temperature on slopes of 2% MEA, and crosses were then set up in 9 cm Petri dishes containing 25 mL oat meal agar (OMA) as described by Ashton and Dyer [39]. This included a preliminary screen of the effect of incubation temperature between 28–37 °C on crossing success. In main crossing efforts, the worldwide isolates of *A. fumigatus* were crossed with four tester strains, two of each mating type, chosen from the original population from Dublin, Ireland because they were the most sexually fertile in terms of number of cleistothecia produced [19]. These four tester isolates were *MAT1-1* strains 47-51 and 47-59 (synonyms AFIR974 and AFRB2, respectively) and *MAT1-2* strains 47-52 and 47-55 (synonyms AFIR964 and AFIR928, respectively; note that 47-55 was one of the supermater strains of Sugui et al. [24]. Three replicate crosses were set up for each cross, with worldwide isolates of known *MAT1-1* genotype being crossed with the *MAT1-2* tester strains, and *MAT1-2* genotypes being crossed with the *MAT1-1* tester strains, in addition to control crosses being set up between the Irish *MAT1-1* and *MAT1-2* tester strains. Crosses were incubated for four months, and sexual fertility was assayed by using a dissecting microscope to carefully inspect plates and then count the number of cleistothecia produced per 9 cm Petri dish, using a hoovering technique to ensure that all cleistothecia could be detected [24,39]. Where crosses were found to be infertile, crosses were incubated for up to 12 months in total before a final inspection (plates were resealed as necessary to prevent drying out of media). In subsequent crossing efforts, crosses involving mating partners other than those from Ireland were set up to verify the global potential for sexual recombination using the same crossing procedures.

### 2.4. Heat Shock, Viability and Germination of Ascospores

Ascospore viability was determined based on their ability to germinate and form hyphal colonies. A lactophenol cotton blue staining method was used to inspect individual ascospores and emergent colonies, which was considered more accurate than relying on applying aliquots of known spore dilutions, which can inherently have some variability and inaccuracy. Growth conditions were as identified by Swilaiman [38] as follows. Ascospores were isolated and 50,000 spores per mL suspensions were prepared as previously described [19,39]. 50 µL aliquots were transferred to sterile 0.2 mL PCR tubes and heat shocked for 30 min at either 65 °C, 75 °C, 80 °C or 85 °C to kill any contaminating conidia. 10 μL aliquots were then spread over marked 2.0 cm diameter circular areas of thinly poured 9 cm ACM plates and incubated at 37 °C for 14 h. Two replicate plates were set up per test cross, with a total of six circular areas marked. To accurately assess percent germination, a drop of lactophenol cotton blue [40] was then added to each marked circular area allowing visualisation of ascospores and arising colonies, which at the same time stopped any further hyphal growth. Counts of germinating ascospores were made using an Olympus light microscope at ×400 magnification. A total of 100 ascospores were evaluated per circle, counting the number of both germinating and nongerminating ascospores, resulting in a total of 600 ascospores being evaluated per cross.

### 2.5. Supermater and Pyrimidine Auxotrophic Strains

During the course of studies, certain supermater strains with high sexual fertility were identified. To further enhance their possible use as community tools, attempts were made to establish pyrimidine auxotrophic derivatives utilising 5’ fluoroorotic acid (5-FOA) selection as used elsewhere for *Aspergillus* species [41,42,43,44]. Conidial suspensions (1 × 10^7^ spores/mL) were prepared in 0.05% Tween-80 and 100 μL aliquots were pipetted onto ACM plates containing 1.2 g/L of both uridine and uracil, and 1.0 mg/L 5-FOA. Plates were incubated at 28 °C for 3–5 days. Emergent colonies were selected and subcultured on ACM slopes supplemented with 1.2 g/L of both uridine and uracil. Pyrimidine auxotrophy was confirmed by subculturing onto ACM plates without uridine/uracil supplementation and onto GG10 minimal media [44], and onto 5-FOA plates to confirm resistance. It has been reported that pyrimidine auxotrophy can influence the sexual fertility of *Aspergillus* species [45,46]. To assess fertility of the resulting pyrimidine auxotrophic strains, the sexual cycle was, therefore, induced on OMA plates supplemented with four different levels of both uridine and uracil (0.3, 0.6, 1.2 and 1.8 g/L of each chemical), using conditions described above [39], and numbers of cleistothecia formed per cross were scored after 3–4 months incubation.

## 3. Results

### 3.1. A. fumigatus Worldwide Collection and Mating-Type Distribution

A total of 131 isolates of *A. fumigatus* were obtained from six continents, of which 111 were from an environmental source and 20 from clinical specimens (Supplemental Table S1). This included 77 new isolates collected for the present study. RAPD-PCR finger-printing revealed that six isolates shared an identical fingerprint pattern to one other isolate and were deemed likely to be clonal samplings. Amplicons were successfully obtained from the remaining 128 isolates using the multiplex PCR diagnostic test of Paoletti et al. [22]. This revealed an almost exact 1:1 ratio of mating types with 52% being *MAT1-1* (*n* = 66) and 48% being *MAT1-2* (*n* = 62) (Table 1). For most regions where more than one isolate was available, both mating types were present (Supplemental Table S1). However, isolates from some regions were only of one mating type, for example in Vietnam where the four isolates obtained were all of *MAT1-1* genotype.

### 3.2. Sexual Fertility of Global A. fumigatus Isolate Collection

**Figure 1:**
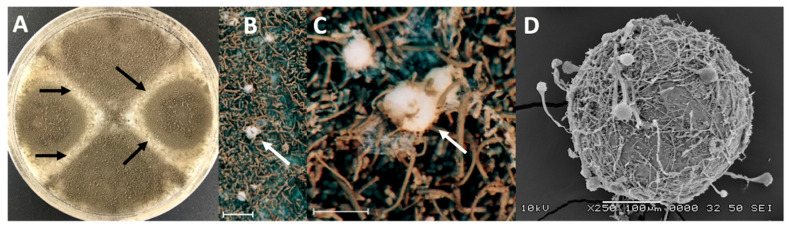
Production of cleistothecia by *A. fumigatus* in crosses with Irish tester strains. (**A**–**C**) Cleistothecia (arrowed) formed along the junctions (barrage zones) of intersecting colonies of opposite mating type. Scale bars = 1 cm. (**D**) Scanning electron micrograph of a cleistothecium showing attachment of superficial conidiophores. (Images A and D courtesy of George Ashton and Paul Brett, University of Nottingham).

**Figure 2:**
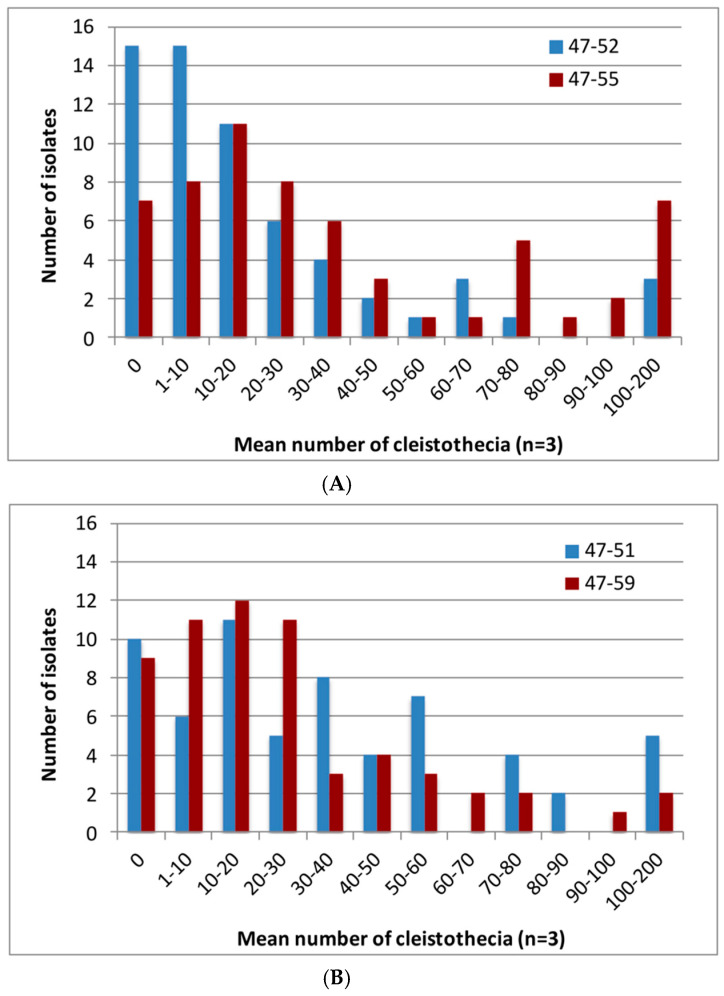
Variation in mean number of cleistothecia produced in crosses with different worldwide isolates of *A. fumigatus*. (**A**) Numbers of cleistothecia produced by *MAT1-1* global isolates (per 9 cm Petri dish), in crosses with two Irish *MAT1-2* tester strains (47-52, 47-55). (**B**) Numbers of cleistothecia produced by *MAT1-2* global isolates (per 9 cm Petri dish), in crosses with two Irish *MAT1-1* tester strains (47-51, 47-59). Left hand axis shows the number of isolates producing the mean number of cleistothecia shown on axis below.

A total of 262 crosses were set up between Irish reference strains and isolates from other worldwide locations using an incubation temperature of 30 °C in darkness, which was shown to be near optimal in preliminary crossing work (the optimum temperature varied between 30–34 °C according to specific cross; Supplemental Figure S1). It was found that, overall, 83.5% of the 131 worldwide isolates produced cleistothecia by mating with the Irish tester strains after four months of incubation on OMA (Figure 1A–D), comprised of 82% of *MAT1-1* isolates and 85% of *MAT1-2* isolates (Figure 2A,B). Thus, a key finding was that sexual fertility was not restricted to the Irish isolates but was present in isolates worldwide. However, the degree of fertility, in terms of cleistothecia formed, varied considerably according to the crossing partners. There were highly statistically significant differences between *MAT1-1* isolates in numbers of cleistothecia produced when they were crossed to the *MAT1-2* Irish reference strains (47-52 and 47-55) (Wilcoxon test *z* = −4.244, *p* < 0.001). Most crosses (83 and 67%, respectively) produced fewer than 50 cleistothecia per crossing plate. However, a minority of crosses (7 and 13% for 47-52 and 47-55, respectively) produced more than 100 cleistothecia (Figure 2A; Supplemental Table S2). Similarly, when worldwide *MAT1-2* isolates were crossed with the *MAT1-1* Irish tester strains (47-51 and 47-59), most crosses (65 and 79%, respectively) produced fewer than 50 cleistothecia and only a minority of crosses (10 and 4%, respectively) produced more than 100 cleistothecia per plate (Figure 2B; Supplemental Table S2). There were again statistically significant differences in numbers of cleistothecia produced in these crosses (Wilcoxon test *z* = −3.663, *p* < 0.001).

There was no clear impact of geographic distance from the Irish reference strains on the fertility of crosses. The highest number of cleistothecia were consistently formed by *MAT1-1* and *MAT1-2* isolates from Zimbabwe (47-187 and 47-190, respectively), *MAT1-1* isolates from India and China (47-235 and 47-169, respectively), and *MAT1-2* isolates from California and the UK (47-239 and 47-154, respectively) (Supplemental Table S2). Even when crossing to other European isolates, a wide variation in fertility was observed with the Irish strains, from sterile through to highly fertile crosses (Supplemental Tables S1 and S2). The degree of fertility was also observed to depend upon the Irish partner strain, with tester strains 47-55 and 47-51 consistently producing more cleistothecia than strains 47-52 and 47-59 (Figure 2A,B). There was no significant difference in fertility between *MAT1-1* and *MAT1-2* isolates from environmental sources, or between *MAT1-2* isolates from environmental and clinical sources (Mann-Whitney test: *z* = −037, *p* = 0.971). However, unexpectedly, *MAT1-1* clinical isolates were significantly more fertile than clinical *MAT1-2* isolates, and there was a significant increase in the number of cleistothecia formed by *MAT1-1* clinical isolates compared to environmental isolates (Mann-Whitney test *z* = −2.103, *p* = 0.036). This might have been an artefact of the relatively small number of clinical isolates assessed. It is noted that among the crosses attempted with the Irish strains were mating studies with two isolates, 47-5 (=Af293) and 47-102 (=A1163, derived from CEA10) which are the first two isolates of *A. fumigatus* for which fully annotated genomes are available, and whose derivatives have been widely used in laboratory studies [21,47,48,49]. All attempts to cross 47-5 failed, whilst crosses with 47-102 showed only relatively low levels of fertility (Supplemental Table S2). Later attempts to mate a KU^80^ CEA17 mutant (also derived from CEA10) similarly resulted in very low fertility or sterile crosses [50].

### 3.3. Sexual Fertility of Isolates Other than from Ireland

Following the success of the crossing efforts involving the Irish tester strains, further sets of crosses were made between a subset of the *MAT1-1* and *MAT1-2* global isolates of *A. fumigatus*. The aim was to determine if sexual reproduction was possible between isolates of opposite mating type from sites other than Ireland, i.e., to verify that fertility was not dependent on an Irish mating partner. Twelve representative isolates (six *MAT1-1* and six *MAT1-2*) were chosen from different continents and countries worldwide and were crossed in all combinations (Table 2). Cleistothecia were formed in the majority of crosses, demonstrating that sexual reproduction is possible between worldwide isolates of *A. fumigatus.* However, it was found that crosses varied significantly in their degree of fertility (one-way ANOVA F = 14.82; df = 1, 34; *p* = 0.008). Certain isolates produced cleistothecia in all crosses (such as the *MAT1-1* isolates from China, India, and South Africa) whereas other isolates (such as the *MAT1-1* isolates from Germany and Hawaii) exhibited consistently low fertility (Table 2).

Given that certain isolates exhibited consistently high fertility, further crosses were set up with 20 supposedly infertile isolates of *A. fumigatus* (eight *MAT1-1* and 12 *MAT1-2*) which had failed to produce cleistothecia with the Irish tester strains. All of the infertile *MAT1-1* isolates were then found to produce cleistothecia when crossed with the highly fertile *MAT1-2* isolate from Zimbabwe (47-190) (Supplemental Tables S3 and S4). Most of the infertile *MAT1-2* strains were also able to produce cleistothecia with at least one crossing partner, although three isolates remained infertile (Supplemental Table S4). Thus, combining the earlier results with the Irish crosses, cleistothecia were produced by 97% of the 131 global isolates tested.

### 3.4. Influence of Heat Shock on Ascospore Germination

Ascospores were recovered from five different crosses (47-215 × 47-59, 47-214 × 47-52, 47-173 × 47-51, 47-170 × 47-51, and 47-110 × 47-55) and used in preliminary heat shock treatments. The heat shock temperature was found to have a highly significant effect on activating ascospore germination (two-way nonparametric ANOVA; H = 43.20, df = 3, *p* < 0.001) (Supplemental Figure S2). Highest germination rates were observed following heat shock at 80 °C for 30 min (overall mean of 47.4 ± 15.7% for the five crosses). By contrast, less than 10% germination occurred following exposure to 65 °C, indicating that high temperature shock was needed to break dormancy, and at 85 °C ascospores from two crosses (47-215 × 47-59 and 47-114 × 47-52) did not appear to survive the heat treatment. Therefore, heat shock at 80 °C for 30 min was chosen to assay ascospore germination.

### 3.5. Ascospore Viability and Germination

**Figure 3:**
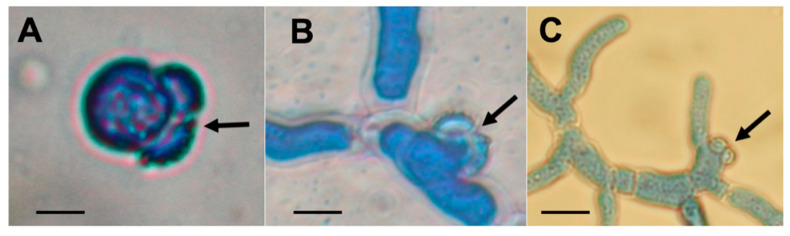
Images of different stages of ascospore germination and emergent colony formation by *A. fumigatus* following staining with lactophenol cotton blue. Arrows indicate opening of bifurcate ascospores with remnants of outer wall shell visible. Scale bars: (**A**), (**B**) = 5 µm; (**C**) = 10 µm.

**Figure 4:**
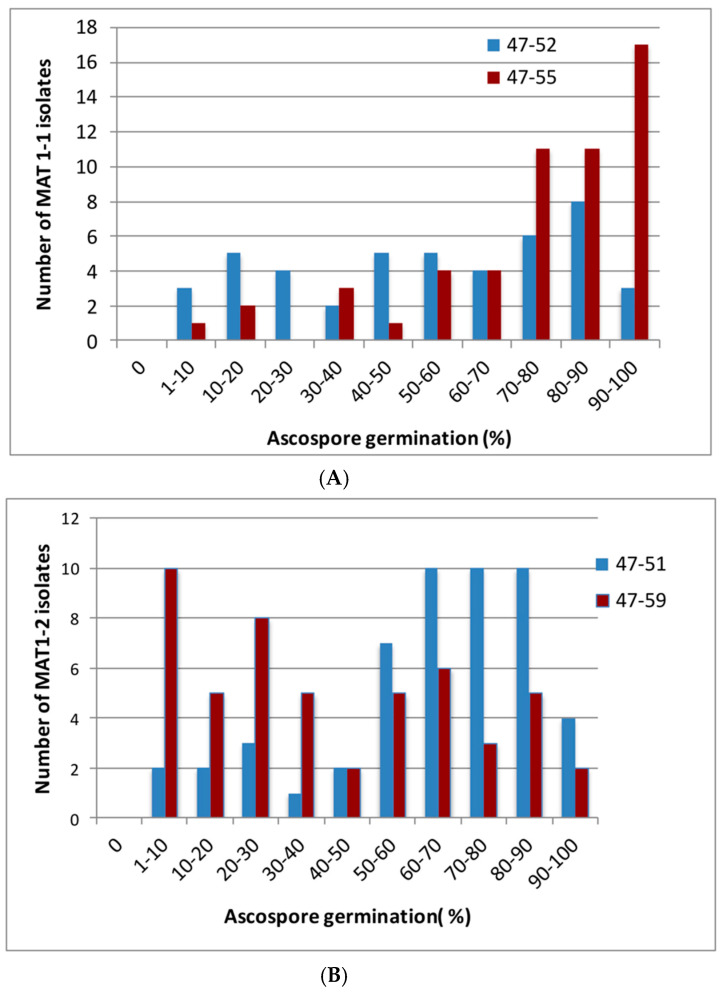
Variation in ascospore germination rate from crosses between different worldwide isolates of *A. fumigatus* and Irish tester strains. (**A**) Germination rates of ascospores produced when *MAT1-1* global isolates were crossed to two *MAT1-2* Irish tester strains (47-52 and 47-55). (**B**) Germination rates of ascospores produced when *MAT1-2* global isolates were crossed to two *MAT1-1* Irish tester strains (47-51 and 47-59). Left hand axis shows the number of isolates producing the bands of rates (1–10%, 10–20% etc.) of ascospore germination shown on axis below.

Ascospores were found to be present in representative cleistothecia in all the fertile crosses between the Irish tester strains and isolates from other worldwide locations. When ascospore germination was inspected using the lactophenol cotton blue staining method (Figure 3) an important overall result was that all crosses were found to form viable ascospores. The majority of crosses produced ascospores with >50% germination rates, and all crosses produced ascospore progeny with at least 1–10% germination under the assay conditions. However, there was very wide variation in rates of germination, with values between 1–98% observed (Figure 4A,B; Supplemental Table S2), with a statistically highly significant variation in germination rates between ascospores arising from crosses between different *MAT1-1* isolates and the tester *MAT1-2* isolates (Wilcoxon test *z* = −4.169, *p* < 0.001), and separately between ascospores arising from crosses between different *MAT1-2* isolates and the tester *MAT1-1* isolates (Wilcoxon test *z* = −4.199, *p* < 0.001).

Notably, different Irish mating partners generated ascospores with different rates of germination. When global *MAT1-1* isolates were crossed to the *MAT1-2* Irish tester strains, 87% of isolates crossed with 47-55 yielded ascospores with more than 50% germination, whereas just 58% of isolates crossed with 47-52 yielded more than 50% ascospore germination (Figure 4A). Similarly, in crosses between the global *MAT1-2* isolates and the Irish *MAT1-1* tester strains, 79 % of crosses with 47-51 yielded more than 50% germination, whereas just 41% of crosses with 47-59 yielded more than 50% germination (Figure 4B). Overall, there was a slightly higher ascospore germination rate from crosses involving the global *MAT1-1* isolates compared to crosses with the global *MAT1-2* isolates, but the difference was not statistically significant (one way ANOVA; F = 3.40; df = 1, 258; P = 0.0664).

**Figure 5:**
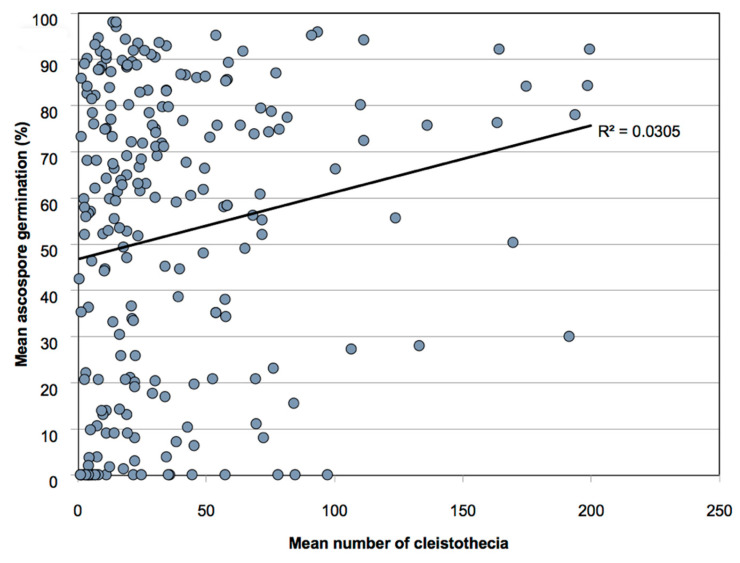
Relationship between the number of cleistothecia formed in crosses of *A. fumigatus* and germination rate of ascospores from the respective cleistothecia. R^2^ =coefficient of determination.

Attempts were made to assess any correlation between the extent of ascospore germination and the number of cleistothecia formed in crosses to see whether more fertile crosses (in terms of number of cleistothecia formed) had higher or lower rates of ascospore germination. However, no correlation was evident (Figure 5).

### 3.6. New Supermater and Fertile Pyrimidine Auxotrophic Strains

In the main and subsequent crossing efforts, certain highly fertile strains, which formed cleistothecia containing viable ascospores within 4–8 weeks, were identified resembling the supermater strains of Sugui et al. [24]. Notably the *MAT1-1* strain 47-169 and the *MAT1-2* strain 47-190 reliably formed cleistothecia with a wide variety of global isolates in comparable or higher numbers than the existing *MAT1-1* and *MAT1-2* supermater strains AFB62 (47-267) and AFIR928 (47-55), respectively, of Sugui et al. [24] (Table 2; Supplemental Tables S2, S5 and S6) and are here offered for community use as additional supermater strains. To enhance possible use (particularly given the low fertility of the widely used CEA10 strain and its CEA17 and A1163 derivatives) 5-FOA selection of these and the existing supermater strains [24] was performed, together with isolates 47-51, 47-187, 47-236 and 47-239, which were also found to produce relatively high numbers of cleistothecia. At least one stable pyrimidine auxotrophic strain was obtained per isolate. Crosses were then set up with tester isolates 47-51 (*MAT1-1*) and 47-190 (*MAT1-2*). It proved possible to obtain over 100 cleistothecia per plate on OMA, supplemented with varying levels of uridine and uracil, with certain of the pyrimidine auxotrophic strains, notably the *MAT1-1* strain 47-267-1 (derivative of *MAT1-1* supermater strain AFB62), and the *MAT1-2* strains 47-236-1 and 47-239-12 (Supplemental Figure S3). However, the optimum level of uridine/uracil varied per cross, and pyrimidine auxotrophic strains of other isolates either showed much reduced sexual fertility or were sterile. For example, it was not possible to induce sexual reproduction in auxotrophs of the new supermater strain 47-190 (Supplemental Figure S3). Pyrimidine auxotrophy has elsewhere been reported to influence sexual fertility in *A. nidulans* [45,46]. The precise nature of mutation in the pyrimidine auxotrophs is currently being investigated. This likely involves mutation in the promoter region of the *pyrG* gene and awaits complementation studies with an intact *pyrG* including a functional promoter region [W. Du, M. Brock and P. S. Dyer, Personal Communication].

## 4. Discussion

The discovery of a sexual cycle in *A. fumigatus* (teleomorph *Neosartorya fumigata*) by O’Gorman et al. [19] had many potential implications for the understanding of the population biology and evolution of this important opportunistic pathogen. For example, if sexual recombination were a regular occurrence in nature, this could allow the spread of genes linked to antifungal resistance and virulence through populations with the risk of producing superfit progeny containing combinations of medically adverse genes [26]. In addition, the meiotic process itself may lead to the evolution of increased antifungal resistance, with reports of progeny of *A. fumigatus* and the plant pathogen *Occulimacula* (*Tapesia*) *yallundae* being generated through the sexual cycle with increased resistance to azole antifungals compared to the parental strains, possibly due to unequal cross over or other meiotic events [27,51]. However, the study of O’Gorman et al. [19] only demonstrated sexual fertility in a small sample of environmental isolates of *A. fumigatus* from a population around Dublin, Ireland, which already showed evidence of recombination. Therefore it has remained unclear to what extent global populations of *A. fumigatus* retain the ability to undergo sexual reproduction, especially given that there is increasing evidence of a slow decline or loss of sexual fertility and evolution towards asexuality in other fungal human and plant pathogens such as Cryptococcus, Fusarium, Magnaporthe and Rhynchosporium species [26,31,52,53,54,55,56].

A key result of the present study was, therefore, that approximately 84% of a global collection of 131 *A. fumigatus* isolates from six continents were able to produce cleistothecia when crossed with Irish *MAT1-1* and *MAT1-2* tester strains from the original study of O’Gorman et al. [19]. This shows that sexual fertility is a general characteristics of *A. fumigatus* worldwide, rather than being restricted to a subset of isolates such as the population from Dublin, Ireland or from the other limited regions. Furthermore, subsequent crossing efforts showed that isolates from different continents were able to cross among themselves and produce cleistothecia, showing that crossing was not reliant on an Irish mating partner. Indeed, it later proved possible to induce the formation of cleistothecia in isolates that had failed to mate with the Irish tester strains (when these were crossed to alternative worldwide strains) meaning that, overall, 97% of isolates tested were sexually competent. These results are consistent with earlier studies of Szewczyk & Krappmann [30] who reported successful crossing of two clinical isolates of *A. fumigatus* unrelated to the Irish environmental isolates of O’Gorman et al. [19], Sugui et al. [24] who were able to obtain cleistothecia in crosses between 50 strains mainly of clinical origin in the USA, and Camps et al. [25] who were able to cross a set of Dutch clinical isolates of different CSP type.

Although almost all worldwide isolates of *A. fumigatus* in the present study were able to produce cleistothecia in crosses, a very wide spectrum of sexual fertility was seen in terms of the number of cleistothecia formed, both in the original crosses to the Irish tester strains and in subsequent crosses between global isolates. Some crosses produced over 200 cleistothecia per 9 cm agar plate, whereas many other crosses produced less than 10 cleistothecia per plate. This suggests differences in the potential for genetic exchange between strains in nature, with the fertility of crosses hard to predict. There was no clear geographic distinction evident in terms of the extent of fertility. For example, isolates from both China and Zimbabwe varied from very low to high fertility, although this observation would need to be confirmed with more in-depth regional sampling and crossing. These results were similar to previous reports of crossing of *A. fumigatus* [19,24] and other pezizomycete species, where a wide variation in number of ascomata were produced in crosses, including related heterothallic species such as *A. flavus*, *A. lentulus*, *A. parasiticus*, and *Neosartoya udagawae* [37,57,58,59]. Thus, compatibility at the mating-type locus (*MAT1-1* or *MAT1-2*) is not the only determinant of sexual fertility, and it remains to be elucidated what may make an isolate a highly fertile supermater or sterile. Other factors such as variable expression or mutation in genes required for sexual reproduction, improper regulation of *MAT* genes, variable synthesis of sex hormones, the presence of secondary sexual incompatibility and possible mismatch in karyotype or cytoplasmic factors have been suggested to explain variations in the degree of fertility of isolates [60,61,62,63].

In the present study repeated vegetative subculture also might have resulted in a decrease in sexual fertility in certain isolates [26], although efforts were made to minimise subculture of freshly obtained isolates. The crossing method used might also have introduced artefacts, as the oatmeal agar used might not have been optimal for certain isolates of *A. fumigatus*, noting that the substrate/location in nature for sexual reproduction remains unknown, although it has been speculated that the sexual cycle might occur in warm decaying compost [19,27]. Indeed, different forms of oatmeal can impact on the sexual fertility of crosses, with rolled oatmeal appearing to promote sexual fertility [24] (Supplemental Figure S4). Interestingly, Lim and Park [64] recently described a method termed vegetative mass mating in which mycelial balls of the different mating types were coinoculated onto oatmeal agar, which was claimed to increase the numbers of cleistothecia produced. A novel mating-type assay for *A. fumigatus*, based on loop-mediated isothermal amplification (LAMP) technology, has recently been described for *A. fumigatus* [65]. Nevertheless, even the most highly fertile isolates of *A. fumigatus* in the present study exhibited relatively low fertility compared to other related heterothallic, as well as homothallic, Neosartorya species such as *N. fennelliae*, *N. nishmurae*, *N. spathulate* and *N. otanii* which produce cleistothecia too numerous to count [66]. However, the findings concerning *A. fumigatus* are similar to studies on *A. parasiticus*, *A. flavus*, *N. udagawae*, and *A. lentulus* which show relatively low levels of fertility [37,57,58,59,66]. It has been suggested that one of the reasons for the disparity in sexual fertility might be that the latter fastidious species compensate by their proficiency to reproduce asexually in a wide range of environmental conditions [2,66].

When ascospores were recovered from cleistothecia produced in the crosses with the Irish *MAT1-1* and *MAT1-2* tester strains and their viability checked, a key result was that all crosses produced viable ascospores that germinated to form mycelial colonies, i.e., gene flow was possible between global isolates of *A. fumigatus* with no absolute postzygotic barrier. However, a very wide spectrum of viability was seen, matching the variation seen in terms of cleistothecia formed, ranging from less than 10% to almost 100% germination rates among ascospores collected from different crosses. These results again indicated likely differences in the potential for genetic exchange between strains. Such high variation in germination rate was unexpected, and the underlying reason for such differences is unclear. There have been very few studies measuring ascospore germination rates as an indicator of sexual fertility, most studies instead focusing on fertility in terms of number of ascomata formed. One exception was the study of Dettman et al. [67] who found that ascospore germination rates were generally >50% in intraspecies crosses of Neurospora but were lower in interspecies crosses. Thus, genetic incompatibility factors might influence the success of germination [60]. Indeed, there is recent evidence of population genetic structuring of global isolates of *A. fumigatus*, with different reports suggesting between eight and two subgroups of *A. fumigatus* [15,68,69]. Therefore, both the variation in viability of ascospores, and the earlier described variation in numbers of cleistothecia produced in crosses, might be due, in part, to differential mating success when crossing different subgroups of *A. fumigatus*. However, no clear correlation was evident between the extent of ascospore germination and the number of cleistothecia formed, i.e., crosses producing higher numbers of cleistothecia did not necessarily have higher rates of ascospore germination.

Regarding the ascospore viability work, it was shown that ascospores of *A. fumigatus* required a heat shock to break dormancy and induce ascospore germination, with less than 10% germination following exposure to 65 °C compared to an almost five-fold increase following heat shock at 80 °C for 30 min. Ascospores of related Neosartorya species, and fungi such as *Talaromyces macrosporus,* Neurospora and Byssochlamys species, also require a heat trigger to break dormancy [29,70,71]. The fact that a heat shock was needed for ascospores of *A. fumigatus* to germinate is, in itself, medically and ecologically significant. If ascospores require such a high temperature heat shock to germinate, they might not act as infectious agents, although other physiological conditions in the human host might trigger germination. Meanwhile, the ecological significance is less clear given the apparent rarity of such extreme temperatures and the fact that most other Aspergillus species produce ascospores with only moderate heat resistance. However, it is conceivable that the cell walls of the *A. fumigatus* ascospores might degenerate over time to allow, for example, the relatively high temperatures found in some rotting vegetation heaps to break the dormancy of these ascospores. Alternatively, certain nutrient triggers or other environmental stress conditions such as osmotic or pH stress, might induce germination. Indeed, the variation in ascospore germination rates seen in the present study might be due, in part, to different crosses producing ascospores with different cell wall thicknesses and solute contents [29], hence with different sensitivity to the heat shock applied under the assay conditions used.

One final output of the present work was the identification of a limited number of highly fertile *MAT1-1* and *MAT1-2* strains of *A. fumigatus*, which were able to produce high numbers of cleistothecia with isolates from different global sites, normally within a 4–8 week period. Sugui et al. [24] have already described the identification of two such supermater strains (*MAT1-1*: AFB62 = 47-267 and *MAT1-2*: ARIR928 = 47-55), so the newly identified supermater strains (*MAT1-1*: 47-169 from China and *MAT1-2*: 47-190 from Zimbabwe) complement those already available and might allow crossing of isolates from nature that otherwise fail to cross with the original supermater strains. In the present study we also generated sexually fertile *MAT1-1* and *MAT1-2* pyrimidine-requiring auxotrophic strains for community use, given that strains with such nutrient requirement markers can provide valuable genetic tools [41,42,43,44]. Isogenic *MAT1-1* and *MAT1-2 A. fumigatus* strains derived from the original supermater strains are already available [72], and a further pair have recently been developed [G. Ashton and P. S. Dyer, Unpublished Data]. Further ΔakuB derivative strains to allow efficient gene deletion targeting [73] are currently in development given the very low fertility or sterility of currently available ΔakuB strains of *A. fumigatus* ([50]; W. Du, M. Brock and P. S. Dyer, Personal Communication).

## 5. Conclusions

In conclusion it is shown that isolates of *A. fumigatus* from many different countries, and from both clinical and environmental sources, have the ability to undergo sexual reproduction. Furthermore, the majority of crosses yielded ascospores exhibiting >50% germination. Although levels of sexual fertility under the assay conditions were relatively low compared to many other fungi, it has been pointed out that “a little sex can go a long way” [74]. Indeed, the fact that a number of surveys have found near 1:1 ratios of the two mating types is consistent with the occurrence of sex maintaining a balance of mating types [19,22,31,75,76], together with evidence of sexual recombination based on STRAf typing at local, regional and global scales [68]. Therefore, the possibility of sexual reproduction is of high medical significance and needs to be accounted for in disease management given the possible emergence and spread of resistance to antifungal drugs [15,25] and implications for diagnostic tests based on the assumption of clonality. Further work is now required to assess the extent of sexual fertility between the recently identified subgroups of *A. fumigatus* described by Ashu et al. [68] and Sewell et al. [69].

## Figures and Tables

**Table 1:** Source and number of worldwide isolates of *A. fumigatus*, and distribution (%) of mating type (*MAT1-1* or *MAT1-2*) in different continents.

Source	Number	*MAT1-1*	*MAT1-2*
Europe	39	61.5%	38.5%
North America	29	48.3%	51.7%
South America	7	42.9%	57.1%
Asia	27	55.6%	44.4%
Africa	23	43.5%	56.5%
Australia	3	33.3%	66.7%

**Table 2:** Number of cleistothecia produced (per 9 cm Petri dish) in crosses between six isolates of *MAT1-1* and six isolates of *MAT1-2* genotype of *A. fumigatus* * from different continents and countries worldwide. Crossing results with Irish tester strains are included for comparison. Numbers are mean of three replicates ± SD.

			*MAT1-2*						
			Asia	Africa		South America	North America		Europe
***MAT1-1***			**India**	**South Africa**	**Zimbabwe**	**Brazil**	**USA** **(San Francisco)**	**USA** **(Michigan)**	**Ireland**
	**Asia**	**India**	36 ± 7	81 ± 57	104 ± 61	29 ± 19	77 ± 47	15 ± 4	33 ± 28
		**China**	24 ± 10	113 ± 37	192 ± 14	180 ± 13	126 ± 22	98 ± 18	111 ± 34
	**Africa**	**South Africa**	99 ± 12	2 ± 3	11 ± 14	65 ± 22	118 ± 26	25 ± 13	72 ± 30
	**North America**	**Hawaii**	0	0	0	7 ± 6	26 ± 5	0	72 ± 28
	**Europe**	**Portugal**	0	0	174 ± 84	6 ± 10	19 ± 14	0	22 ± 12
		**Germany**	0	0	24 ± 18	0	0	0	19 ± 26
		**Ireland**	77 ± 77	25 ± 24	317 ± 78	32 ± 3	46 ± 66	84 ± 36	58 ± 12

* *MAT1-1* isolates used were from India (47-147), South Africa (47-159), China (47-169), Portugal (47-122), Germany (47-212), Hawaii (47-180) and Irish strain 47-51. *MAT1-2* isolates used were from India (47-142), South Africa (47-140), Zimbabwe (47-190), Brazil (47-135), USA-San Francisco (47-107), USA-Michigan (47-215) and Irish strain 47-55.

## Supplementary Materials

The following are available online at https://www.mdpi.com/2309-608X/6/4/258/s1, Figure S1: Effect of incubation temperature between 28–37 °C on production of cleistothecia on two representative crosses of *A. fumigatus*. Figure S2: Mean percentage germination (%) of ascospore suspensions from five different crosses of *A. fumigatus* following exposure to 65 °C, 75 °C, 80 °C, or 85 °C heat treatment for 30 min. Figure S3: Effect of supplementation of various levels of uridine and uracil on production of cleistothecia in crosses between tester *MAT1-1* and *MAT1-2* strains of *A. fumigatus* and various pyrimidine auxotrophic strains. Figure S4: Effect of different types of oatmeal agar (Odlums Pinhead Oatmeal from Ireland, Quaker Old Fashioned Rolled Oats from USA, Traditional Rolled Oats from UK) on production of cleistothecia on two representative crosses of *A. fumigatus*. Table S1: Details of *A. fumigatus* isolates used in sexual crossing experiments, including their *MAT* locus genotype, country of origin, isolation site and year of isolation. Table S2: Excel sheet showing number of cleistothecia produced by *MAT1-1* and *MAT1-2* worldwide isolates of *A. fumigatus* in crosses with Irish *MAT1-2* (47-52, 47-55) and *MAT1-1* (47-51, 47-59) tester strains, respectively. Data sheet also includes details of the % ascospore germination per cross where available. Table S3: Number of cleistothecia produced by *MAT1-1* isolates of *A. fumigatus* which were infertile in crosses with Irish tester strains, when crossed to different high fertility global *MAT1-2* mating partners. Table S4: Number of cleistothecia produced by *MAT1-2* isolates of *A. fumigatus* which were infertile in crosses with Irish tester strains, when crossed to different high fertility global *MAT1-1* mating partners. Table S5: Number of cleistothecia produced by different *MAT1-1* and *MAT1-2* isolates of *A. fumigatus* after four-week incubation on oatmeal agar. Table S6: Number of cleistothecia produced by different *MAT1-1* and *MAT1-2* isolates of *A. fumigatus* after eight weeks incubation on oatmeal agar.
