# Supplementary material for: Global Sexual Fertility in the Opportunistic Pathogen Aspergillus fumigatus and Identification of New Supermater Strains
Source: J Fungi (Basel). 2020 Oct 30;6(4):258. doi: 10.3390/jof6040258 (PMC7712211; doi:10.3390/jof6040258)
Supplement: Supplementary file 1 [file jof-06-00258-s001.zip › jof-985738-supplementary/Supplemental files_/JoF Supp Figure S1.docx]

**Supplemental Figure S1.** Figure showing effect of incubation temperature between 28 – 37 ºC on production of cleistothecia on two representative crosses of *Aspergillus fumigatus*, 47-248 x 47-154 and 47-259 x 47-236. Crosses were scored after 4 weeks growth on oatmeal agar in 9 cm Petri dishes (n=4), error bars represent ±SEM.
