# Supplementary material for: Global Sexual Fertility in the Opportunistic Pathogen Aspergillus fumigatus and Identification of New Supermater Strains
Source: J Fungi (Basel). 2020 Oct 30;6(4):258. doi: 10.3390/jof6040258 (PMC7712211; doi:10.3390/jof6040258)
Supplement: Supplementary file 1 [file jof-06-00258-s001.zip › jof-985738-supplementary/Supplemental files_/JoF Supp Figure S2.docx]

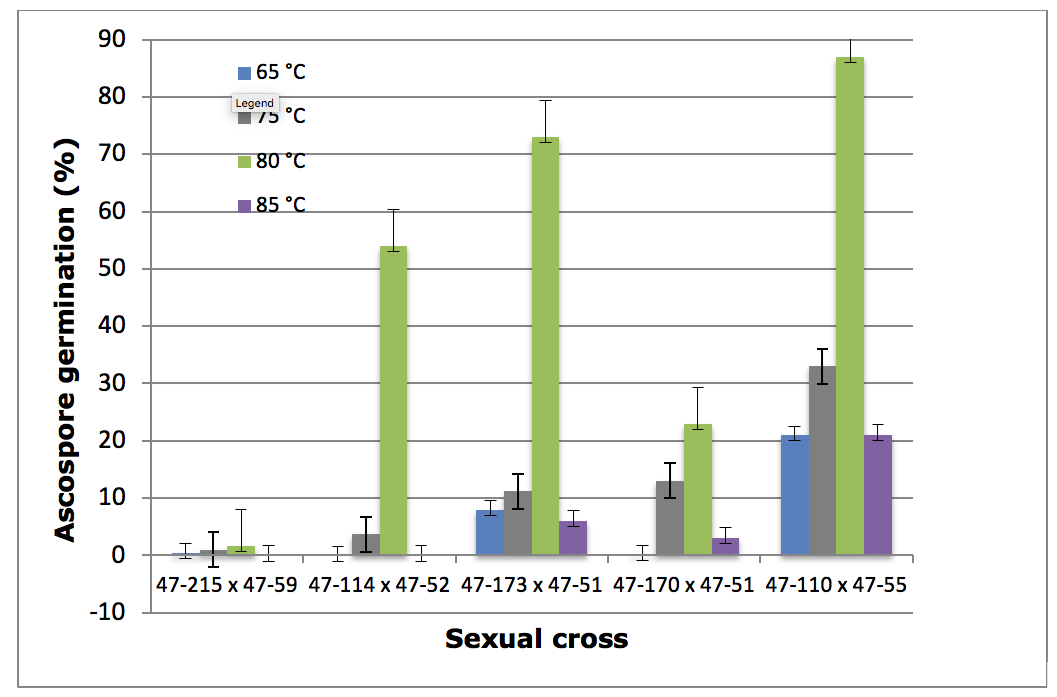


**Supplemental Figure S2.** Figure showing mean percentage germination (%) of ascospore suspensions from five different sexual crosses of *Aspergillus fumigatus* following exposure to 65°C, 75 °C, 80°C, or 85 °C heat treatment for 30 min, and then incubation at 37 °C for 14 h. Error bars refer to ± SEM.
