# Supplementary material for: Global Sexual Fertility in the Opportunistic Pathogen Aspergillus fumigatus and Identification of New Supermater Strains
Source: J Fungi (Basel). 2020 Oct 30;6(4):258. doi: 10.3390/jof6040258 (PMC7712211; doi:10.3390/jof6040258)
Supplement: Supplementary file 1 [file jof-06-00258-s001.zip › jof-985738-supplementary/Supplemental files_/JoF Supp Figure S3.docx]

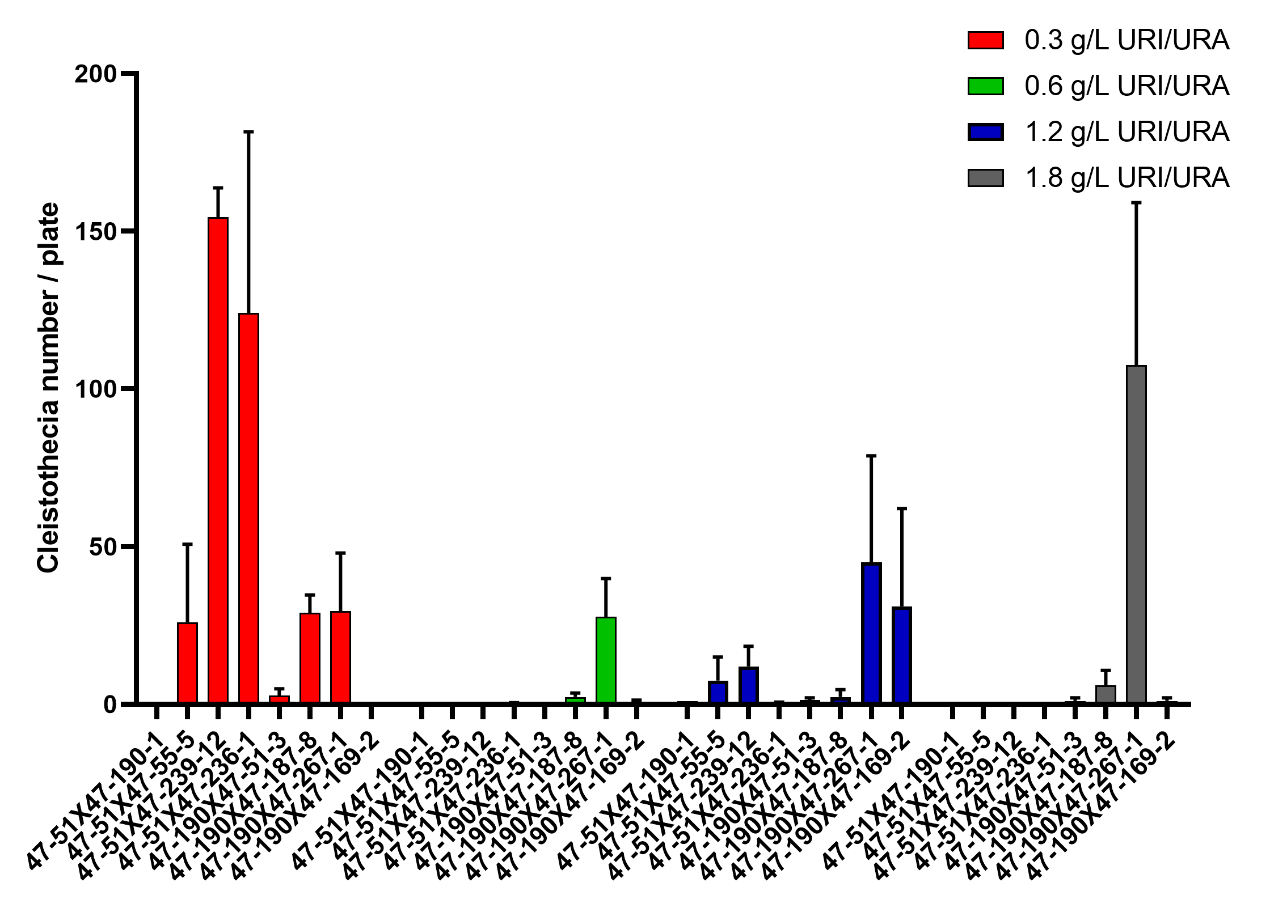


**Supplemental Figure S3.** Figure showing the effect of supplementation of various levels of both uridine and uracil (0.3, 0.6, 0.9 and 1.8 g/l of each chemical) on production of cleistothecia in crosses between *Aspergillus fumigatus* tester isolates 47-51 (*MAT1-*1) and 47-190 (*MAT1-2*) and various representative pyrimidine auxotrophic strains. Numbers indicate average number of cleistothecia produced per 9 cm Petri dishes (n=4) containing oat meal agar; error bars represent ±SEM. Crosses were scored after 8 wk growth in darkness at 30 ºC.
