# Supplementary material for: Global Sexual Fertility in the Opportunistic Pathogen Aspergillus fumigatus and Identification of New Supermater Strains
Source: J Fungi (Basel). 2020 Oct 30;6(4):258. doi: 10.3390/jof6040258 (PMC7712211; doi:10.3390/jof6040258)
Supplement: Supplementary file 1 [file jof-06-00258-s001.zip › jof-985738-supplementary/Supplemental files_/JoF Supp Figure S4.docx]

**Supplemental Figure S4.** Figure showing effect of different types of oatmeal agar (made with either Odlums Pinhead Oatmeal from Ireland, Quaker Old Fashioned Rolled Oats from USA, or Traditional Rolled Oats from UK) on production of cleistothecia on two representative crosses of *Aspergillus fumigatus*, 47-248 x 47-154 and 47-259 x 47-236. Crosses were scored after 4 weeks growth on oatmeal agar in 9 cm Petri dishes (n=7) at 30 ˚C in the dark. The horizontal black bars indicate significantly different means (***P* ≤ 0.01; ****P* ≤ 0.001). Coloured bars represent the mean; error bars represent ± one SEM.
