# Supplementary material for: Global Sexual Fertility in the Opportunistic Pathogen Aspergillus fumigatus and Identification of New Supermater Strains
Source: J Fungi (Basel). 2020 Oct 30;6(4):258. doi: 10.3390/jof6040258 (PMC7712211; doi:10.3390/jof6040258)
Supplement: Supplementary file 1 [file jof-06-00258-s001.zip › jof-985738-supplementary/Supplemental files_/JoF Supp Table S1.docx]

##

## **Supplemental Table S1.** Details of *Aspergillus fumigatus* isolates used in sexual crossing experiments, including their *MAT* locus genotype, country of origin and isolation site, where known.

| **Nottingham**  **Number (BDUN)*** | **Original Strain**  **Number^†^** | **Source^‡^** | **Alternative**  **Code Numbers^†^** | **Country** | **City/location^¶^** | **Isolation Site^§^** | ***MAT* locus^∫^** |
| --- | --- | --- | --- | --- | --- | --- | --- |
| 47-1 | AF50 | E | ATCC 34625 | USA | Maryland | Balsa Wood | *1-2* |
| 47-2 | AF1 | C | ATCC 201531 | USA | Palo Alto, SUH, California | Pleural fluid (1988) | *1-1* |
| 47-3 | AF62 | E | M3021 | USA | Palo Alto, SUH, California | Lab contaminant | *1-1* |
| 47-4 | AF250 | C | FA/201 | UK | Salford, HH. Manchester | ― | *1-1* |
| 47-5 | AF293 | C | CBS 101355 | UK | Shrewsbury, Shropshire | Lung biopsy  (1993) | *1-1* |
| 47-6 | AF41 | C | 88-130 | USA | Palo Alto, SUH, California | Pericardial tissue | *1-2* |
| 47-7 | AF217 | E | ― | Sweden | ― | Sawmill A | *1-1* |
| 47-8 | AF10 | C | ATCC 90240 | USA | Los Angeles | Lung aspirate  (1986) | *1-2* |
| 47-9 | AF70 | E | AF54 | USA | Camden, New Jersey | Air sample | *1-2* |
| 47-10 | AF221 | E | ― | New Zealand | ― | Sawmill A | *1-2* |
| 47-49 | CBS 133.61 | C | ATCC 1022 | USA | Connecticut | Chicken Lung  (1961) | *1-2* |
| 47-51 | AFIR974 | E | ― | Ireland | Dublin | Air sampling  (2005) | *1-1* |
| 47-52 | AFIR964 | E | ― | Ireland | Dublin | Air sampling  (2005) | *1-2* |
| 47-55 | AFIR928 | E | ― | Ireland | Dublin | Air sampling  (2005) | *1-2* |
| 47-59 | AFRB2 | E | ― | Ireland | Dublin | Air sampling  (2005) | *1-1* |
| 47-102 | FGSC A1163 | C | CBS 144.89  CEA10, A1163 | France | Paris | Man (1989) | *1-1* |
| 47-103 | TUBF-11 | E | MMRL2684 | Italy | Pisa | Soil (1982) | *1-2* |
| 47-104 | TUBF-18 | E | MMRL2685 | Thailand | Bangkok | Soil (1983) | *1-2* |
| 47-105 | TUBF-32 | E | MMRL2686  ATCC 58128 | Hungary | Badacsony | Grape compost  (1983) | *1-2* |
| 47-106 | TUBF-55 | E | MMRL2687 | Jamaica | Jamaica Town | Soil (1983) | *1-2* |
| 47-107 | TUBF-266 | E | MMRL2688 | USA | San Francisco area | Soil (1988) | *1-2* |
| 47-108 | TUBF-275 | E | ― | USA | Downey, Idaho | Lava flow soil  (1988) | *1-1* |
| 47-109 | TUBF-439 | E | MMRL2690 | Tunisia | Cartago, Carthago | Soil (1983) | *1-1* |
| 47-110 | TUBF-440 | E | MMRL2691 | Portugal | Covina, Coina | Soil (1985) | *1-1* |
| 47-111 | ATCC42824 | E | MMRL2853 | Hungary | Budapest | Organic fertilizer  (1977) | *1-1* |
| 47-112 | ATCC42826 | E | MMRL2854 | Hungary | Szarvas | Fallen cherry leaf  (1978) | *1-2* |
| 47-113 | TUBF-44 | E | MMRL2856 | Hungary | Badacsony | Sunflower seed hull compost (1983) | *1-1* |
| 47-114 | TUBF-87 | E | MMRL2855  ATCC 58129 | Hungary | Badacsony | Sunflower seed hull compost (1983) | *1-1* |
| 47-116 | TUBF-113 | E | MMRL2858 | Hungary | Badacsony | Sewage  compost (1985) | *1-2* |
| 47-117 | TUBF-115 | E | MMRL3859 | Hungary | Badacsony | Sewage  compost (1985) | *1-1* |
| 47-118 | TUBF-265 | E | MMRL2842 | USA | San Francisco area | Soil (1988) | *1-2* |
| 47-119 | TUBF-345 | E | MMRL2843 | Hungary | Kiskunhalas | Water pipeline  Industrial water (1990) | *1-1* |
| 47-120 | TUBF-435 | E | MMRL2844 | Brazil | Sao Paulo | Soil (1991) | *1-1* |
| 47-121 | TUBF-437 | E | MMRL2845 | Japan | Tokyo | Soil (1985) | *1-2* |
| 47-122 | TUBF-441 | E | ― | Portugal | Coina | Soil (1985) | *1-1* |
| 47-123 | TUBF-442 | E | MMRL2847 | Italy | Rome | Soil (1989) | *1-2* |
| 47-124 | TUBF-1385 | E | MMRL2848 | Russia | Ufa | Soil (1992) | *1-2* |
| 47-125 | TUBF-1387 | E | MMRL2849 | Russia | Ufa | Soil (1992) | *1-2* |
| 47-126 | TUBF-1388 | E | MMRL2850 | Zimbabwe | Masamba | Soil (2002) | *1-2* |
| 47-127 | TUBF-1389 | E | ― | Zimbabwe | Masamba | Soil (2002) | *1-2* |
| 47-128 | TUBF-1390 | E | MMRL2852 | Zimbabwe | Masamba | Soil (2002 | *1-2* |
| 47-129 | TUBF-1426 | E | ― | Brazil | Salvador/Bahia | Soil (1997) | *1-2* |
| 47-130 | TUBF-1427 | E | ― | Brazil | Salvador/Bahia | Soil (1997) | *1-1* |
| 47-131 | TUBF-1428 | E | MMRL3040 | South Africa | Natal | Soil (2002) | *1-1* |
| 47-132 | TUBF-1429 | E | ― | South Africa | Natal | Soil (2002) | *1-2* |
| 47-134 | TUBF-1431 | E | MMRL3054 | Brazil | Manaus | Soil (1997) | *1-1* |
| 47-135 | TUBF-1432 | E | MMRL3053 | Brazil | Manaus | Soil (1997) | *1-2* |
| 47-136 | TUBF-1433 | E | MMRL3052 | India | Trivandrum | Soil (1997) | *1-2* |
| 47-137 | TUBF-1434 | E | MMRL3051 | South Africa | Empangeni | Soil (2002) | *1-1* |
| 47-138 | TUBF-1435 | E | MMRL3049 | South Africa | Umkomaas | Soil (2002) | *1-2* |
| 47-139 | TUBF-1436 | E | MMRL3050 | South Africa | Umkomaas | Soil (2002) | *1-2* |
| 47-140 | TUBF-1437 | E | MMRL3061 | South Africa | Umkomaas | Soil (2002) | *1-2* |
| 47-142 | TUBF-1450 | E | ― | India | Trivandrum | Soil (1997) | *1-2* |
| 47-143 | TUBF-1451 | E | ― | India | Trivandrum | Soil (1997) | *1-1* |
| 47-144 | TUBF-1452 | E | ― | India | Agra | Leaf compost (1997) | *1-1* |
| 47-145 | TUBF-1453 | E | ― | India | Agra | Leaf compost (1997) | *1-1* |
| 47-146 | TUBF-1454 | E | ― | India | Agra | Leaf compost (1997) | *1-2* |
| 47-147 | TUBF-1455 | E | ― | India | Agra | Leaf compost (1997) | *1-1* |
| 47-148 | TUBF-1456 | E | ― | India | Agra | Leaf compost (1997) | *1-1* |
| 47-149 | TUBF-1468 | E | MMRL3070 | Sudan | Khartoum | (1984) | *1-2* |
| 47-150 | TUBF-1469 | E | MMRL3071 | Australia | New South Wales | Soil (1988) | *1-2* |
| 47-151 | TUBF-1470 | E | MMRL3072 | Australia | New South Wales | Soil (1988) | *1-1* |
| 47-152 | TUBF-1471 | E | MMRL3073 | India | Agra | Leaf compost (1997) | *1-2* |
| 47-153 | TUBF-1472 | E | MMRL3074 | India | Agra | Leaf compost (1997) | *1-1* |
| 47-154 | ― | E | ― | UK | Sutton Bonington, Nottingham | Straw (2012) | *1-2* |
| 47-155 | TUBF-1569 | E | ― | South Africa | Cape Town | Soil (2001) | *1-1* |
| 47-156 | TUBF-1570 | E | ― | South Africa | Cape Town | Soil (2001) | *1-2* |
| 47-157 | TUBF-1571 | E | ― | South Africa | Cape Town | Soil (2001) | *1-1* |
| 47-158 | TUBF-1572 | E | ― | South Africa | Cape Town | Soil (2001) | *1-2* |
| 47-159 | TUBF-1573 | E | ― | South Africa | Cape Town | Plant compost  (2001) | *1-1* |
| 47-160 | TUBF-1574 | E | ― | South Africa | Cape Town | Plant compost  (2001) | *1-2* |
| 47-161 | TUBF-1575 | E | ― | Vietnam | Ho Chi Minh  (Saigon) | Soil (2001) | *1-1* |
| 47-162 | TUBF-1576 | E | ― | Vietnam | Ho Chi Minh  (Saigon) | Soil (2001) | *1-1* |
| 47-163 | TUBF-1577 | E | ― | Vietnam | Ho Chi Minh  (Saigon) | Soil (2001) | *1-1* |
| 47-164 | ― | E | ― | UK | Sutton Bonington, Nottingham | Straw (2012) | *1-1* |
| 47-169 | TUBF -1771 | E | ― | China | Shanghai | Soil (2004) | *1-1* |
| 47-170 | TUBF-1772 | E | ― | China | Shanghai | Soil (2004) | *1-2* |
| 47-171 | TUBF-1773 | E | ― | China | Hangzhou | Soil (2004) | *1-2* |
| 47-172 | TUBF-1774 | E | ― | China | Hangzhou | Soil (2004) | *1-2* |
| 47-173 | TUBF-1814 | E | ― | Zambia | Lusaka | Soil (1983) | *1-2* |
| 47-174 | TUBF-1815 | E | ― | Brazil | Sao Paulo | Soil (1991) | *1-2* |
| 47-175 | TUBF-1816 | E | ― | Brazil | Sao Paulo | Soil (1991) | *1-2* |
| 47-180 | TUBF-1839 | E | ― | Hawaii | Oahu | Soil (1997) | *1-1* |
| 47-181 | TUBF-1840 | E | ― | Hawaii | Oahu | Soil (1997) | *1-1* |
| 47-187 | TUBF-1901 | E | ― | Zimbabwe | Harare | Soil (2002) | *1-1* |
| 47-188 | TUBF-1578 | E | ― | Vietnam | Ho Chi Minh  (Saigon) | Soil (2002) | *1-1* |
| 47-189 | TUBF-1858 | E | ― | Sri Lanka | Hill Country | Soil (1991) | *1-1* |
| 47-190 | TUBF-1910 | E | ― | Zimbabwe | Harare | Soil (2002) | *1-2* |
| 47-191 | TUBF-1857 | E | ― | Sri Lanka | Hill Country | Soil (1991) | *1-1* |
| 47-192 | TUBF-1902 | E | ― | Zimbabwe | Harare | Soil (2002) | *1-2* |
| 47-193 | TUBF-1859 | E | ― | Sri Lanka | Garden | Soil (1991) | *1-1* |
| 47-212 | NRRL-164 | E | ATCC 1028  CBS 113.26 | Germany | ― | Soil (1926 or earlier?) | *1-1* |
| 47-213 | NRRL1979 | E | ATCC 9197  QM 6858 | USA | New Jersey | Soil (1943) | *1-2* |
| 47-215 | NRRL 2869 | E | ATCC 34475 | USA | Michigan | Soil (late 1950s) | *1-2* |
| 47-218 | TUB F-2436 | E | ― | USA | New Orleans | Soil (1996) | *1-2* |
| 47-219 | TUB F-2437 | E | ― | USA | New Orleans | Soil (1996) | *1-1* |
| 47-220 | TUB F-2438 | E | ― | USA | New Orleans | Soil (1996) | *1-1* |
| 47-222 | TUB F-2443 | E | ― | Mauritius | ― | Soil (2000) | *1-1* |
| 47-223* | TUB F-2444 | E | ― | Mauritius | ― | Soil (2000) | *1-1* |
| 47-224* | TUB F-2445 | E | ― | Mauritius | ― | Soil (2000) | *1-1* |
| 47-225 | TUB F-2449 | E | ― | Mexico | Mexico City | Decaying plant (2005) | *1-2* |
| 47-226 | TUB F-2450 | E | ― | Mexico | Mexico City | Decaying plant (2005) | *1-1* |
| 47-227* | TUB F-2451 | E | ― | Mexico | Mexico City | Decaying plant (2005) | *1-2* |
| 47-228* | TUB F-2452 | E | ― | Mexico | Mexico City | Decaying plant (2005) | *1-2* |
| 47-229 | TUB F-2453 | E | ― | Mexico | Mexico City | Decaying plant (1999) | *1-1* |
| 47-230 | TUB F-2455 | E | ― | USA | Anaheim, California | Soil (2005) | *1-1* |
| 47-231 | TUB F-2456 | E | ― | USA | Anaheim, California | Soil (1999) | *1-1* |
| 47-232 | TUB F-2457 | E | ― | USA | Anaheim, California | Soil (1999) | *1-1* |
| 47-233 | TUB F-2458 | E | ― | Cuba | Matanzas | Soil (1999) | *1-1* |
| 47-234 | TUB F-2459 | E | ― | India | Poona | Soil (1998) | *1-2* |
| 47-235 | TUB-2460 | E | ― | India | Poona | Soil (1998) | *1-2* |
| 47-236 | - | E | ― | UK | Sutton Bonington, Nottingham | Straw (2012) | *1-2* |
| 47-237 | TUB F-2462 | E | ― | India | Trivandrum | Soil (1998) | *1-2* |
| 47-238 | TUB F-2463 | E | ― | India | Trivandrum | Soil (1998) | *1- 1* |
| 47-239* | TUB F-2464 | E | ― | USA | Hollywood, California | Soil (1999) | *1- 2* |
| 47-240 | TUB F-2465 | E | ― | USA | Hollywood, California | Soil (1999) | *1- 2* |
| 47-241* | TUB F-2466 | E | ― | USA | Hollywood, California | Soil (1999) | *1- 2* |
| 47-242 | TUB F-2467 | E | ― | Cuba | Matanzas | Soil (1999) | *1-1* |
| 47-246 | 09w414118 | C | ― | UK | Mansfield | Sputum (2009) | *1-2* |
| 47-247 | 09W414820 | C | ― | UK | Nottingham | Bronchial lavage  (2009) | *1-1* |
| 47-248 | 09W414118 | C | ― | UK | Nottingham | Bronchial lavage  (2009) | *1-1* |
| 47-249 | 10w404187 | C | ― | UK | Nottingham | Sputum (2010) | *1-1* |
| 47-250 | 09w416071 | C | ― | UK | Newark | Sputum (2009) | *1-2* |
| 47-251 | 10w400721 | C | ― | UK | Newark | Sputum (2010) | *1-2* |
| 47-253 | 10w401558 | C | ― | UK | Nottingham | Sputum (2010) | *1-2* |
| 47-254 | 09w414598 | C | ― | UK | Mansfield | Sputum (2009) | *1-1* |
| 47-255 | 09w412001 | C | ― | UK | Nottingham | Lung Biopsy (2009) | *1-1* |
| 47-256 | 09W402655 | C | ― | UK | Nottingham | Sputum  (2009) | *1-1* |
| 47-257 | ― | C | ― | UK | Nottingham | N/A | *1-1* |
| 47-258 | 09w414372 | C | ― | UK | Nottingham | Sputum (2009) | *1-1* |
| 47-259 | 10w401716 | C | ― | UK | Nottingham | Sputum (2010) | *1-1* |

**^*^**Denotes possible clonal isolates (applies to 47-223 and 47-224; 47-227 & 47-228; and 47-239 and 47-241).

^†^AF=University of Manchester (UK); AFIR/AFR = University of Dublin (Ireland); ATCC= American Type Culture Collection; CBS= Centraal Bureau voor Schimmelcultures (Westerdijk Institute, The Netherlands); FGSC= Fungal Genetics Stock Centre (USA); NRRL= Northern Regional Research Laboratory (USA); TUBF= Technical University of Budapest (Hungary); 09/10w=Queens Medical Centre, Nottingham (UK).

**^‡^**C and E indicate isolates from clinical and environmental sources, respectively.

**^¶^**SUH= Stanford University Hospital; HH= Hope Hospital.

**^§^**Year of collection in parentheses where known. Note that TUB-F soil samples were stored refrigerated at 5-8 ºC prior to isolation and lyophilisation, in some cases 5-10 years later.

**^∫^**According to multiplex PCR *MAT* diagnostic of Paoletti et al. [21].
