# Supplementary material for: Global Sexual Fertility in the Opportunistic Pathogen Aspergillus fumigatus and Identification of New Supermater Strains
Source: J Fungi (Basel). 2020 Oct 30;6(4):258. doi: 10.3390/jof6040258 (PMC7712211; doi:10.3390/jof6040258)
Supplement: Supplementary file 1 [file jof-06-00258-s001.zip › jof-985738-supplementary/Supplemental files_/JoF Supp Table S5.docx]

**Supplemental Table S5.** Numbers of cleistothecia (mean ± standard deviation, *n*=4) produced in crosses involving different *MAT1-1* and *MAT1-2* isolates of *Aspergillus fumigatus* after incubation at 30 °C for 4 wk on Oat Meal Agar plates in the dark.

| Cross | | *MAT1-1* | | | | |
| --- | --- | --- | --- | --- | --- | --- |
|  |  | AFB62 (47-267) | 47-259 | 47-169 | 47-235 | 47-248 |
| *MAT1-2* | 47-55  (AFIR928) | 15.8 ± 5.3 | 8.3 ± 9.9 | 83.0 ± 57.1 | 0.5 ± 1.0 | 23.5 ± 33.0 |
|  | 47-190 | 31.0 ± 43.9 | 0.5 ± 1.2 | 4.0 ± 6.1 | 0 | 21.8 ± 37.6 |
|  | 47-236 | 22.3 ± 11.5 | 0 | 37.8 ± 72.9 | 5.8 ± 11.5 | 0 |
|  | 47-154 | 2.3 ± 4.5 | 1.0 ± 2.0 | 13.0 ± 21.0 | 0 | 0 |
|  | 47-239 | 17.8 ± 35.5 | 0 | 5.3 ± 6.8 | 0 | 0 |
