# Supplementary material for: Global Sexual Fertility in the Opportunistic Pathogen Aspergillus fumigatus and Identification of New Supermater Strains
Source: J Fungi (Basel). 2020 Oct 30;6(4):258. doi: 10.3390/jof6040258 (PMC7712211; doi:10.3390/jof6040258)
Supplement: Supplementary file 1 [file jof-06-00258-s001.zip › jof-985738-supplementary/Supplemental files_/JoF Supp Table S6.docx]

**Supplemental Table S6.** Numbers of cleistothecia* produced in crosses involving different *MAT1-1* and *MAT1-2* isolates of *Aspergillus fumigatus* after incubation at 30 °C for 8 wk on Oat Meal Agar plates in the dark.

| Cross | | *MAT1-1* | |
| --- | --- | --- | --- |
|  |  | 47-259 | 47-169 |
| *MAT1-2* | 47-190 | ++++  ++++  ++++  ++++ | ++++  +++  +++  +++ |
|  | 47-154 | +  ++  NF  + | +++  ++  +++  ++ |
|  | 47-142 | +++  ++++  +++  + | +  +  ++  ++ |
|  | 47-115 | ++  ++  +  + | +++  ++++  ++++  ++++ |
|  | 47-236 | ++  ++  +  ++ | +++  N/A  N/A  N/A |
|  | 47-239 | N/A  N/A  N/A  N/A | +  +  +  + |
|  | 47-240 | ++  +  ++  N/A | +++  +++  ++  ++ |
|  | 47-250 | +  ++  +  NF | +++  ++  +  +++ |

*NF = Not fertile; + = <50 cleistothecia; ++ = <100 cleistothecia; +++ = >100 cleistothecia; ++++ = >200 cleistothecia. N/A = Not available. Refers to average number of cleistothecia formed per 9 cm Petri dish.
