# Supplementary material for: Global Sexual Fertility in the Opportunistic Pathogen Aspergillus fumigatus and Identification of New Supermater Strains
Source: J Fungi (Basel). 2020 Oct 30;6(4):258. doi: 10.3390/jof6040258 (PMC7712211; doi:10.3390/jof6040258)
Supplement: Supplementary file 1 [file jof-06-00258-s001.zip › jof-985738-supplementary/Supplemental files_/JoF Supp Tables S3 and S4.docx]

**Supplemental Table S3.** Number of cleistothecia produced by *MAT1-1* isolates of *Aspergillus fumigatus,* (which were infertile in crosses with Irish tester strains), in crosses with different high fertility global *MAT1-2* mating partners on oatmeal agar medium at 30˚C in the dark after six months.

| Crosses | | Number of cleistothecia^*^ | | |
| --- | --- | --- | --- | --- |
|  |  | ***MAT1-2*** | | |
|  |  | 47-236 | 47-239 | 47-190 |
| ***MAT1-1*** | 47-3 | - | - | ++ |
|  | 47-4 | - | - | ++ |
|  | 47-151 | + | - | + |
|  | 47-153 | - | - | + |
|  | 47-224 | - | - | + |
|  | 47-229 | - | - | + |
|  | 47-233 | - | - | + |
|  | 47-242 | - | - | + |

^*^Ratings indicate the mean number of cleistothecia produced from three replicate crosses on oatmeal agar in 9 cm diameter Petri dishes after incubating in the dark for six month at 30 °C: -, none; +, 1-19; ++, 20-39.

**Supplemental Table S4.** Number of cleistothecia produced by *MAT1-2* isolates of *Aspergillus fumigatus* (which were infertile in crosses with Irish tester strains), in crosses with different high fertility global *MAT1-1* mating partners on oatmeal agar medium at 30˚C in the dark after six months.

| Crosses | | Number of cleistothecia^*^ | |
| --- | --- | --- | --- |
|  |  | ***MAT1-1*** | |
|  |  | 47-169 | 47-259 |
| ***MAT1-2*** | 47-1 | + | - |
|  | 47-9 | - | - |
|  | 47-134 | + | + |
|  | 47-116 | - | - |
|  | 47-118 | + | + |
|  | 47-124 | - | + |
|  | 47-125 | - | + |
|  | 47-126 | + | -___ |
|  | 47-172 | + | + |
|  | 47-174 | + | - |
|  | 47-225 | + | - |
|  | 47-251 | - | - |

^*^Ratings indicate the mean number of cleistothecia produced from three replicate crosses on oatmeal agar in 9 cm diameter Petri dishes after incubating in the dark for six month at 30 ºC: -none; +, 1-19.
